# Synthesis of Linear Black Gold Nanostructures Processable as Sunlight and Low‐Energy Light Collecting Films for Photo‐Thermoelectricity

**DOI:** 10.1002/advs.202207415

**Published:** 2023-02-24

**Authors:** Jeong Han Kim, Seung Beom Pyun, Min Ju Choi, Ji Won Yeon, Young Ji Hwang, Eun Chul Cho

**Affiliations:** ^1^ Department of Chemical Engineering Hanyang University Seoul 04763 Republic of Korea

**Keywords:** linear black Au nanostructures, low‐energy light collecting films, photo‐thermoelectricity, processibility, winding and bent

## Abstract

As one of the effort to cope with the energy crisis and carbon neutrality, utilization of low‐grade energy generated indoors (e.g., light) is imperative because this saves building and house energy, which accounts for ≈40% of total energy consumption. Although photovoltaic devices could contribute to energy savings, it is also necessary to harvest heat from indoor lights to generate electricity because the light absorbed by materials is mostly transformed into heat. For daily life uses, materials should not only have high absorptance and low emittance but also be easily processed into various forms. To this end, this work synthesizes black aqueous suspensions containing winding and bent linear gold nanostructures with diameters of 3–5 nm and length‐to‐diameter ratios of ≈4–10. Their optical and photo‐thermal characteristics are understood through experimental and theoretical investigations. Black gold nanostructures are conveniently processed into metal‐dielectric films on metal, glass, and flexible substrates. The film on copper has an absorptance of 0.97 and an emittance of 0.08. Under simulated sunlight and indoor LED light illumination, the film has equivalent photo‐thermal and photo‐thermoelectric performances to a top‐tier sunlight‐collecting film. This work attempts to modify the film structure to generate more usable electricity from low‐energy indoor light.

## Graphical abstract

Black aqueous suspensions containing winding and bent linear gold nanostructures are synthesized to prepare various light collecting films (with high absorptance and low emittance) for the production of photo‐thermoelectricty under the sunlight and low‐energy‐light illumination. Particularly, utilization of heats produced from low‐energy lights could save building energy, hence deal with the energy crisis and carbon neutrality.

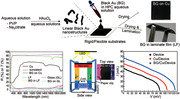

## 1 Introduction

Uncertain supplies of fossil fuels and worldwide initiatives to limit carbon emissions have stimulated research on alternative and clean energy sources. Concurrently, energy savings could be another effective way to deal with these issues. It is particularly crucial to save building and house energy, which accounts for a considerable portion (≈40%) of overall energy use.^[^ ^1^ ^]^ One strategy to achieve this is to convert low‐grade energy (such as light) generated indoors into electricity, because low‐energy light can be found everywhere in houses and office buildings. Light absorption by materials at visible and infrared frequencies is known to either excite electrons to their higher energy levels^[^ ^2^, ^3^ ^]^ or generate hot electrons.^[^ ^4^, ^5^ ^]^ These electrons are useful for photovoltaic devices to generate electricity,^[^ ^2^, ^3^, ^6^, ^7^ ^]^ and so photovoltaic devices might significantly contribute to energy savings by converting building light into electricity. Nonetheless, the development of methods to harvest heat from indoor light is also vital because the majority of light absorbed by materials is converted into heat.^[^ ^8^, ^9^ ^]^ To date, sunlight heat has been mostly used to heat up water and air,^[^ ^10^, ^11^, ^12^ ^]^ and it has recently been employed for water purification and desalination^[^ ^13^, ^14^, ^15^, ^16^ ^]^ as well as to generate electricity using solar thermoelectricity devices.^[^ ^17^, ^18^, ^19^, ^20^, ^21^, ^22^, ^23^, ^24^ **^]^** In addition to the studies with sunlight, it is necessary to explore the usefulness of the thermal energies created from low‐powered lights.

For practical photo‐thermal applications, materials should not only be able to maximally utilize light energies, but should also be easily processed into various forms. Among various materials that produce heat from light, plasmonic materials may be considered as promising candidates because their surface plasmon resonance frequencies mostly lie in the visible and near infrared ranges, which account for ≈90% of total sunlight energy. In addition, most electrical lighting appliances in everyday life emit visible light. The geometries of plasmonic materials can be tailored to tune their resonance frequencies and the values of absorption/scattering cross‐sections (along with the ratios of absorption to extinction cross‐sections).^[^ ^25^, ^26^, ^27^ ^]^ However, to effectively harvest these lights, it is sometimes desirable to design materials that absorb light at broad frequencies. From this standpoint, black gold (Au) nanostructures have received considerable attention in light‐harvesting systems. It has been reported that small Au nanoparticles assemble to form black Au structures.^[^ ^28^, ^29^, ^30^, ^31^ ^]^ Highly branched superparticles are synthesized through the morphological modulation in the seeded growth process.^[^ ^32^ ^]^ Porous or fibrous templates are used to deposit Au or Au nanoparticles through evaporation or wet chemical methods.^[^ ^13^, ^33^, ^34^, ^35^, ^36^ ^]^ Electrochemical methods are used to produce porous structures or particular geometries.^[^ ^37^ ^]^ A lithography technique has also been introduced to create an array of sharp grooves.^[^ ^38^ ^]^ Nonetheless, it is still necessary to develop a convenient method for fabricating/synthesizing structures that can be easily processed into various forms to suit a variety of light‐energy‐harvesting applications.

**Figure 1:**
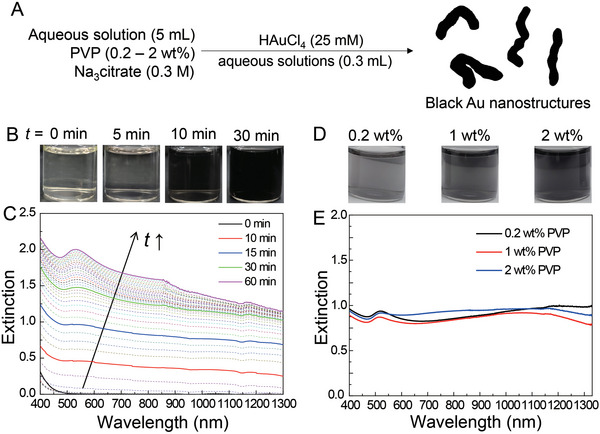
A) A schematic illustrating a typical procedure conducted for the synthesis of the black Au nanostructures. B,C) The change in B) the colors and C) the optical extinction spectra of an aqueous mixture as a function of reaction time (*t*) after the addition of HAuCl_4_ aqueous solution to the aqueous solution containing 0.3 m tri‐sodium citrate and 2 wt% PVP. D) A photograph of the aqueous suspensions synthesized by using the aqueous solution with tri‐sodium citrate and PVP of different concentrations. For all the samples, the reaction was stopped at *t* = 5 min, and the nanostructures were purified by centrifugation and re‐dispersion in deionized water. E) Extinction spectra of the three black Au aqueous suspensions synthesized under the same experimental procedure as described in (D), but the concentrations of the Au nanostructures in the samples were adjusted to compare the spectral shapes among the samples.

Here, we synthesize linear Au nanostructures that can absorb a wide range of visible and near‐infrared light and hence exhibit black Au characteristics. The synthesis protocol is simple (**Figure** **1**A): Adding HAuCl_4_ aqueous solution to an aqueous solution containing tri‐sodium citrate and a water‐soluble polymer results in a black suspension. The linear nanostructures mostly have diameters of 3–5 nm, which lie between those of conventional or mini Au nanorods (≥8 nm)^[^ ^39^, ^40^ ^]^ and ultrathin nanorods (≈2 nm).^[^ ^41^, ^42^ ^]^ Their length‐to‐diameter ratios (aspect ratios, AR) approximately ranged from 4 to 10. Notably, these linear nanostructures are winding and bent. Based on experimental results and literatures, a mechanism underlying the formation of the nanostructures is suggested. The optical properties are understood by comparing the experimental data with the calculated optical spectra. A further theoretical study is performed to investigate the potentiality of the black Au nanostructures to produce heat under light illumination. Black Au nanostructures are processed by mixing with a natural polymer to form a metal‐dielectric composite film on various substrates (metal, glass, and flexible polymers). In addition, the metal‐dielectric film can be laminated with flexible films by introducing a coating machine. We investigate the optical and photo‐thermal characteristics of these films. We also investigate the photo‐thermoelectricity performance of the light‐collecting films on copper (Cu) under simulated sunlight illumination (air mass (AM) 1.5 G) and indoor LED light. The film performances are compared with those of a top‐class light‐collecting film. We further attempt to modify the design and sample geometry to produce more usable electricity from low‐energy indoor light.

## 2 Results and Discussion

### 2.1 Synthesis and Characterization of Black Au Nanostructures

**Figure 2:**
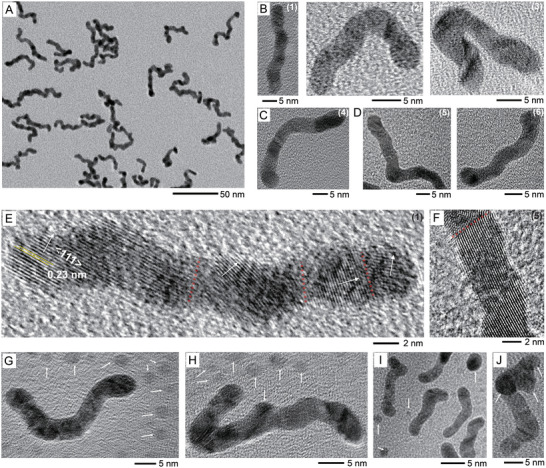
A) A TEM image of the Au nanostructures observed from the black Au aqueous suspension synthesized by using the aqueous solution with tri‐sodium citrate and 0.2 wt% PVP. B–D) TEM images of the individual Au nanostructures observed from the black Au aqueous suspension synthesized by using the aqueous solutions with tri‐sodium citrate and B) 0.2, C) 1, and D) 2 wt% PVP. E,F) Magnified TEM images of the Au nanostructures (1) (in B) and (5) (in D). White arrows indicate the crystal growth direction, and red dashes indicate the boundaries between the two growing Au crystals. G–I) TEM images showing tiny Au nanoparticles (white arrows) around the linear Au nanostructures. J) A TEM image of an Au nanostructure in which tiny nanoparticles appear to be attached to a linear nanostructure. G–J) were observed from the black Au aqueous suspensions synthesized by using the aqueous solutions with tri‐sodium citrate and G,H) 2, I) 1, and J) 0.2 wt% PVP.

The synthesis of linear black Au nanostructures started with the addition of 0.3 mL 25 mm chloroauric acid (HAuCl_4_) aqueous solution to 5 mL aqueous solution containing 0.3 m tri‐sodium citrate and 0.2–2 wt% polyvinylpyrrolidone (PVP). The reaction was carried out at room temperature (20–25 °C). When using an aqueous solution containing tri‐sodium citrate and 2 wt% PVP, for example, the yellowish color due to HAuCl_4_ gradually turned gray and then faint black in 5 min (Figure 1B). The black color became deeper in 10 min and even deeper in 30 min. A closer examination of the suspension revealed that the black color was mixed with a faint plum/purple color. We further investigated the temporal changes in the optical extinction spectra of the aqueous suspension to understand the color change (Figure 1C). The extinction increased with time in the entire visible‐near infrared wavelength range, along with the development of a peak ≈500–600 nm. The peak became significant from the reaction time (*t*) = 30 min and dominant at *t* = 1 h. We attempted to separate the plum/purple colors from the black‐colored suspension (Figure S1A, Supporting Information). The reaction was stopped at *t* = 5 min, and the reaction mass was centrifuged for 30 min at 11 000 rpm. After centrifugation, a black cake formed at the bottom, and the supernatant had a mixture of black and plum/purple color with an extinction peak at ≈530 nm (Figure S1B, Supporting Information). The plum/purple color of the supernatant suspension was more noticeable when comparing to the color of reaction mass before centrifugation (Figure 1B), and hence the reaction was thought to continue during the centrifugation step. The supernatant was removed, and the cake was re‐dispersed in deionized water. The purified aqueous suspension was black in color, as shown in Figure 1D. The color of suspensions was not significantly influenced by the PVP concentration (within the range of 0.2–2 wt%) of the aqueous solution used for this synthesis. Instead, as the PVP concentration increased, the black color of the aqueous suspension became deeper, indicating that the conversion rate of HAuCl_4_ could be influenced by the PVP concentration. The concentrations of the Au nanostructures in the suspension, shown in Figure 1D, were adjusted to compare the spectral shapes of the three black Au aqueous suspensions (Figure 1E). Although there was a slight difference in the spectral shapes of the samples, the three suspensions showed a wide range of light extinction at 500–1300 nm. It is worth noting that there was still a peak ≈515–523 nm, implying that the suspensions, even after purification, still contained Au nanoparticles with different geometries from the black‐colored Au nanostructures, which was further confirmed from the transmission electron microscopy (TEM) images (this will be discussed in **Figure** **2**) and will be further discussed with theoretical studies.

Figure 2A shows a TEM image of Au nanostructures synthesized using the aqueous solution containing tri‐sodium citrate and 0.2 wt% PVP. The sample contained linear nanostructures that were mostly winding and bent. Branched nanostructures were also observed. The black Au aqueous suspension synthesized using the solution with tri‐sodium citrate and 2 wt% PVP had a similar morphology (Figure S2, Supporting Information). Figure 2B shows a few Au nanostructures observed from the black Au aqueous suspension synthesized using the solution with tri‐sodium citrate and 0.2 wt% PVP. Structure (1) was slightly winding with an approximate diameter of 4.1 nm and a length of 33 nm. A winding and slightly bent nanostructure was also found (Figure S3, Supporting Information) with an approximate diameter and fully extended length of 4.6 and 38 nm, respectively. Structure (2) was a bent nanostructure with an approximate bent angle of 90°, and it had an estimated diameter and fully extended length of 3.9 and 28 nm, respectively. Structure (3) was another type of bent nanostructure with a bent angle of 180°, and it had a diameter and extended length of 5 and 34 nm, respectively. The Au nanostructures observed from the black Au aqueous suspension synthesized using the solution with tri‐sodium citrate and 1 or 2 wt% PVP showed similar morphologies ((4)–(6) in Figure 2C,D, also see Figure S4, Supporting Information). From the image analysis of Figure 2 and Figures S2–S4, Supporting Information, the AR was estimated to be mostly in the range between 4 and 10, but some Au nanostructures also had the AR ≥ 10 (from Figure 2A and Figure S2, Supporting Information).

Figure 2E shows a magnified TEM image of the nanostructure (1) shown in Figure 2B. The lattice fringes had an approximate spacing of 0.23 nm, indicating that the {111} facet was exposed to the surface of the Au nanostructure. The single‐crystalline growth was extended to ≈10 nm, but the crystal appeared fused with other crystals with different growth directions (red dashed lines), indicating that the nanostructure had a polycrystalline structure. A similar lattice structure was observed in Figure S3B, Supporting Information (the magnified TEM image of Figure S3A, Supporting Information). The Au nanostructure (5) (Figure 2D) also had a polycrystalline structure, as shown in Figure 2F.

The present synthesis used HAuCl_4_, tri‐sodium citrate, and PVP. Linear Au nanostructures were not obtained without PVP; instead, we observed that tiny Au nanoparticles were aggregated (Figure S5, Supporting Information). These results indicate that tri‐sodium citrate can mainly reduce Au ions to Au(0), and PVP might minimize the aggregation of Au seeds. In addition, because the conversion rate of HAuCl_4_ increased with increasing PVP concentration, we supposed that PVP could also participate in the reduction of Au ions as a mild reducing agent.^[^ ^43^, ^44^ ^]^ We conducted a set of experiment to further investigate the effect of PVP concentration on the morphology of the Au nanostructures. Specifically, the HAuCl_4_ aqueous solution was added to aqueous solutions containing 0.3 m tri‐sodium citrate and PVP of various concentrations. When using the aqueous solution with the PVP concentration of 0.1 wt% (Figure S6, Supporting Information), linear nanostructures were mixed with tiny nanoparticles and nanostructures that were not completely linear. It was estimated that the linear nanostructures mostly had the AR between 2 and 7, shorter than the linear nanostructures synthesized by using the aqueous solutions containing 0.2–2 wt% PVP. With the aqueous solution containing 5 wt% PVP (Figure S7, Supporting Information), we did not observe a distinct difference in the morphologies from those obtained by using the aqueous solutions containing 0.2–2 wt% PVP: the AR values of the linear nanostructures were more or less similar. Meanwhile, for the aqueous solution containing 10 wt% PVP (Figure S8, Supporting Information), the linear Au nanostructures were mixed with the irregular Au nanostructures. It has been reported that PVP can direct the anisotropic growth of noble metal nanostructures^[^ ^45^ ^]^ either by selectively adsorbing on {100} or {111} facets^[^ ^43^, ^46^ ^]^ or by accumulating on plane defects.^[^ ^47^ ^]^ The present experimental results also suggest that PVP could help direct an anisotropic growth of the Au nanostructures. However, the optimum concentration ranges appear to exist to obtain the linear black Au nanostructures.

Several works were reported regarding the synthesis of Au nanowires and nanowire networks based on the sodium citrate and Au precursors in aqueous solutions. Pong et al.^[^ ^48^ ^]^ and Pei et al.^[^ ^49^ ^]^ reported that Au nanowires (networks) were constructed, as intermediate products, at an early reaction stage in the synthesis of conventional spherical Au nanoparticles under boiling or hot reaction conditions. They suggested that the nanowires could be made by linear assemblies of tiny Au nanoparticles (due to the steric effect^[^ ^48^ ^]^), and the event was followed by the deposition of Au atoms on the assembled nanostructures. After the depletion of AuCl_4_^−^, the nanostructures might be disassembled into spherical Au particles. Pei et al. also suggested that the Au nanowires (networks) could be harvested by adjusting the ratio of citrate to Au precursor.^[^ ^49^ ^]^ With a similar mechanism, it was reported that a network of Au nanowires could be synthesized in a basic aqueous conditions.^[^ ^50^ ^]^ In the meantime, a literature introduced oxalate and PVP to synthesize chain‐like network of Au nanowires (diameters ≈15–20 nm):^[^ ^51^ ^]^ it was reported that the oxalate dianion might bridge the Au nanoparticles to assemble in a side‐on fashion for the nanowire formation. This work also mentioned that the PVP controlled the reduction rate of Au precursor by reducing contact between AuCl_4_^−^ and oxalate, and reducing the Au nanoparticle growth.^[^ ^51^ ^]^ Such PVP roles appeared different from the roles observed from our studies. Additionally, there was an earlier literature regarding the production of Au nanowire networks, due to intrinsic anisotropic coalescence of atomic precursor or small Au clusters, from a laser ablation of precleaned flat Au plates in deionized water.^[^ ^52^ ^]^ The literature reported that the average diameters varied from 6 to 12 nm depending on the preparation temperature, the network nanostructures co‐existed with twisted Au nanorods, and the two structures could be separated by a filter process. From these literatures and our experimental results, we understood and speculated that the present winding and bent linear nanostructures could be harvested by capturing one of the early reaction stages of the conventional Au nanoparticle synthesis from the introduction of PVP that could both direct anisotropic growth and minimize/control the aggregation/aggregation states of the Au seeds and nanoparticles.

We further attempted to deduce the mechanism of formation of the polycrystalline linear Au nanostructures, based on the additional experimental results and the literatures. We often observed tiny spherical Au nanoparticles among the linear nanostructures, as shown in Figure 2G,H (see the white arrows). Most spherical particles had diameters ranging from 3 to 5 nm, which is comparable to the diameters of the linear nanostructures, and they appeared to have single‐crystalline structures (Figure S9, Supporting Information). In the other region, spherical Au nanoparticles were mixed with some short linear nanostructures (Figure 2I). Therefore, it is believed that Au(0) was added to the spherical seed nanoparticles to form linear Au nanostructures with the aid of PVP. At the same time, according to the literatures,^[^ ^48^, ^49^ ^]^ some other Au nanoparticles can also attach to the growing nanostructures, resulting in polycrystalline structures. As such case, the growth direction was changeable when the new nanoparticle was not connected with the linear nanostructure by exactly matching with their lattices each other. Consequently, various winding and bent structures can be produced. In some cases, the Au nanoparticles could be attached to the side of the linear Au nanostructures (Figure 2H) or more than two seeds could be attached to the linear nanostructures (Figure 2J and Figure S10, Supporting Information), possibly resulting in branched structures, as shown in Figure 2A.

### 2.2 Optical and Photo‐Thermal Characteristics of Black Au Nanostructures

**Figure 3:**
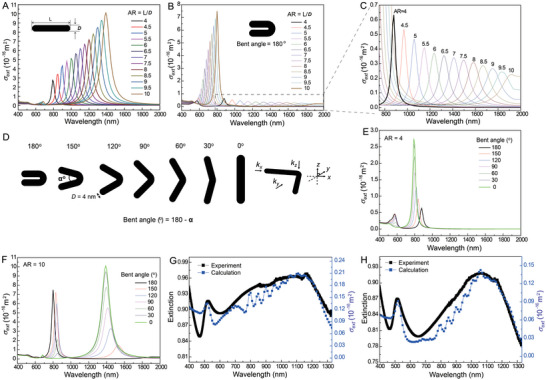
A,B) Calculated extinction cross‐sections (*σ*_ext_) of A) straight and B) bent linear Au nanostructures having a diameter of 4 nm as a function of AR. C) is the magnified spectra of (B). D) Model linear Au nanostructures having a bent angle of 0 to 180° for the calculation of *σ*_ext_. *k_x_, k_y_*, and *k_z_* are the propagation vectors heading *x*‐, *y*‐, and *z*‐directions, respectively. See Experimental Section for more detail. E,F) *σ*_ext_ of the linear Au nanostructures (diameter = 4 nm) having AR of 4 (E) and 10 (F) for various bent angles. G,H) Fitting results (blue squares) to the experimental data (black spheres) of the Au nanostructure aqueous suspensions synthesized by using the aqueous solution with tri‐sodium citrate and G) 2 wt% PVP and H) 1 wt% PVP. The volume fractions of the Au nanostructures were summarized in Table S1, Supporting Information.

We attempted to understand the optical properties of the black‐colored aqueous suspensions containing linear Au nanostructures (Figure 1E) through theoretical studies of straight linear, bent, and winding nanostructures (see Experimental Section for more detail). The COMSOL Multiphysics software was used for the calculations. We first studied the optical spectra of straight linear Au nanostructures with a diameter of 4 nm and varying AR = 4–10 (**Figure** **3**A). The major peaks, owing to their longitudinal resonance mode, were shifted to higher wavelengths with increasing AR from 790 (AR = 4) to 1390 nm (AR = 10). The peak wavelength increase for this resonance mode can also be observed in conventional, mini, and ultrathin nanorods.^[^ ^40^, ^42^, ^53^ ^]^ We investigated the optical spectra of the completely bent nanostructures (U‐shaped) (Figure 3B,C). Two peaks were observed at 570 and 880 nm for AR = 4, and these peaks were shifted to higher wavelengths with increasing AR: they were observed at 800 and 1920 nm, respectively, for AR = 10. The spectral shapes of bent Au nanostructures were similar to those of U‐shaped split‐ring resonators fabricated on substrates.^[^ ^54^, ^55^ ^]^ It was reported that the peak at short and long wavelengths was caused by the electric field component and magnetic resonances of the plasmonic nanostructure, respectively. Importantly, in this study, the peaks generated at short wavelengths generally (for AR = 4.5–10) had higher extinction cross‐sectional values (*σ*_ext_) than those generated at long wavelengths, and these peak wavelengths were shorter than those of the straight linear Au nanostructures (570 nm versus 790 nm for AR = 4 and 800 nm versus 1390 nm for AR = 10). We further investigated the optical spectra of the bent nanostructures by varying the bent angle from 180° (U‐shaped) to 0° (straight) (Figure 3D). As shown in Figure 3E,F, *σ*_ext_ of the peaks shown at shorter wavelengths increased, whereas those shown at longer wavelengths decreased with increasing bent angles. Conclusively, the existence of bent Au nanostructures might mostly contribute to the increase in *σ*_ext_ at visible frequencies, whereas the straight linear Au nanostructures could largely contribute to the increase in *σ*_ext_ at near‐infrared frequencies.

Because the present linear Au nanostructures had various AR and various winding and bent structures (Figure 2), we tried to explain the experimental spectra using the calculated ones. Figure 3G shows the result of the best fit to the experimental spectrum obtained from the black Au suspension synthesized by using the aqueous solution with tri‐sodium citrate and 2 wt% PVP. We briefly summarize the fitting processes and results as follows. A broad spectrum was obtained from ≈750 to 1300 nm when considering only the straight linear Au nanostructures (Figure S11A, Supporting Information); thus, the calculated spectrum did not match the experimental data at visible frequencies. Meanwhile, when considering the bent nanostructures (bent angles of 0–180°), we found that the calculated and experimental spectra were close to each other (Figure S11B, Supporting Information). However, the calculated spectrum exhibited only a small peak at 500–520 nm, and its intensity was not as significant as that of the experimental spectrum. It is general that conventional Au nanorods (mostly having diameter >10 nm) have characteristic peaks ≈500–520 nm, owing to the transverse resonance mode. Meanwhile, it was reported that ultrathin Au nanorods did not show significant cross‐sectional values in the transversal modes compared with the longitudinal resonance modes.^[^ ^42^ ^]^ Therefore, we ascribed the peaks at 500–520 nm from the experiment to the existence of tiny spherical Au (seed) nanoparticles mixed with the linear Au nanostructures, as shown in Figure 2G–I. We calculated the spectra of the spherical Au nanoparticles with diameters of 3, 4, and 5 nm (Figure S11C, Supporting Information), and the peaks were observed ≈515–520 nm. After considering the contribution of the spherical Au nanoparticles, the theoretical calculation results were similar to the experimental values, as shown in Figure 3G. Table S1, Supporting Information, summarizes the estimated volume fractions (%) of linear Au nanostructures and spherical (seed) nanoparticles. We also attempted to match the experimental spectrum of the black Au suspension synthesized by using the aqueous solution with tri‐sodium citrate and 1 wt% PVP, as shown in Figure 3H.

**Figure 4:**
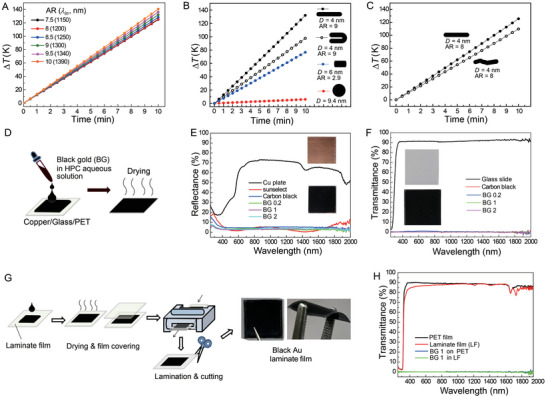
A) Calculated time‐course changes in temperature (*∆T*) of straight linear Au nanostructures (diameter = 4 nm) when a light at a peak wavelength *λ*_in_ (in Figure 3A) is incident on the nanostructure. B) Calculated *∆T* of various Au nanostructures when a light of a peak wavelength *λ*_in_ is incident on the nanostructures: *λ*_in_ were 1300, 760, 680, and 520 nm, respectively, for a straight Au nanostructure, a bent Au nanostructure, a straight Au nanostructure having larger diameter, and an Au nanosphere (diameter = 9.4 nm). They have the same volumes. C) Calculated *∆T* of the straight and winding linear Au nanostructures when a light of a peak wavelength (*λ*_in_ = 1190 and 1200 nm for winding and straight, respectively) is incident on the Au nanostructures. D) A schematic illustrating the fabrication process of the black Au nanostructure films on various substrates. E) Reflectance spectra of a Cu plate, sunselect, HPC‐carbon black, and HPC‐black Au nanostructure films coated on Cu. Photographs: top, the Cu plate; bottom, HPC‐black Au nanostructure films on Cu. F) Transmittance spectra of a glass slide, HPC‐carbon black, and HPC‐black Au nanostructure films coated on the glass. Photographs: top, the glass slide; bottom, HPC‐black Au nanostructure films on the glass. G) A schematic illustrating the fabrication process of the HPC‐black Au nanostructure laminate film (LF). H) Transmittance spectra of a PET, a LF, the HPC‐black Au nanostructure films either coated on the PET or laminated in LF. Abbreviation: BG 0.2, BG 1, BG 2 are the films prepared by using the black Au suspensions synthesized by using the tri‐sodium citrate and 0.2, 1, and 2 wt% PVP, respectively.

It is worth mentioning that the experimental spectra obtained from the aqueous suspensions synthesized using the aqueous solution with tri‐sodium citrate and 0.2 or 1 wt% PVP were slightly different from that of the suspension synthesized using the solution with tri‐sodium citrate and 2 wt% PVP, particularly at wavelengths >1300 nm and intensities at 510–520 nm. From repeated experiments, we found that the difference might not be due to the effect of the PVP concentration used for the synthesis, but that spectra were slightly different from sample to sample, even when using the same concentration of PVP. Therefore, a slight difference was observed during the synthesis and purification processes. Nevertheless, more importantly, all the samples contained similar Au nanostructures (Figure 2), stayed black (from Figure 1), and hence retained similar optical properties when processed into other forms, as will be discussed in **Figure** **4**. It is also worth noting that the bent angles of the linear Au nanostructures were random (and thus not controllable) within the PVP concentration range we used for the synthesis of linear Au nanostructures.

We performed additional calculations as follows. The optical characteristic of the winding linear Au nanostructure modeled based on Figure 2B,E was similar to that of straight linear Au nanostructure (Figure S12A, Supporting Information). However, *σ*_ext_ and the absorption cross‐section (*σ*_abs_) of the winding linear Au nanostructures was slightly lower (*σ*_ext_ = 6.15 × 10^−16^ and *σ*_abs_ = 6.14 × 10^−16^ m^2^) than those of the straight linear Au nanostructure (*σ*_ext_ = 7.17 × 10^−16^ and *σ*_abs_ = 7.15 × 10^−16^ m^2^). The ratios of *σ*_abs_ to *σ*_ext_ for the two nanostructures were almost identical at their peak wavelengths (≈0.997). The maximum extinction peak was slightly shifted to the shorter wavelength (winding: 1190 nm and straight: 1200 nm). Similar result was reported in a literature^[^ ^56^ ^]^ where the absorption and scattering cross section of bent Au nanorods at their longitudinal mode decreased as bent angle increases. In the present calculation, we assumed that the winding structure was constructed by combining the torus segments (bent segments with various bent angles), cylinders (straight segments), and hemispheres. Therefore, *σ*_ext_ and *σ*_abs_ could be smaller than that of straight linear Au nanostructures. Additionally, the peak positions of the straight linear Au nanostructures with a diameter of 4 nm were almost similar to those of Au nanostructures with diameters of 5 nm (Figure S12B, Supporting Information for AR = 6 and 10).

As mentioned above, the calculation results also suggested that the Au nanostructures have an absorption‐to‐extinction ratio of almost 1, implying that almost all the extinct light could theoretically be absorbed by the nanostructures. Because some of the absorbed light can be converted into heat, we theoretically investigated the ability of the linear Au nanostructures to produce heat when they were surrounded by water (see Experimental Section for more details). The straight linear Au nanostructures produced heat under light illumination with wavelengths (*λ*_in_) corresponding to the peaks from their longitudinal modes (Figure 4A). The heating rates increased slightly with increasing AR of the nanostructures. From Figure 4B, the heating rate of the straight linear Au nanostructure (13.2 K min^−1^ for AR = 9) was higher than that of the bent Au nanostructures (9.8 K min^−1^ for AR = 9 and bent angle of 180°), which was in line with a previous report that the bent Au nanorods have lower heating efficiency than the straight Au nanorods.^[^ ^56^ ^]^ Additionally, the heating rates of the straight and bent linear Au nanostructure were higher than that of an Au nanostructure with identical volume but a different dimension (7.7 K min^−1^ for diameter = 6 nm and AR = 2.9) and higher than that of an Au nanosphere having the same volume (0.60 K min^−1^ for diameter = 9.4 nm). From Figure 4C, we found that the winding linear Au nanostructure had a lower heating rate (11.0 K min^−1^ for AR = 8) than that of the straight linear Au nanostructure (12.6 K min^−1^ for AR = 8). Because the photo‐thermal efficiencies could depend on the *σ*_abs_ of Au nanostructures, we understand the photo‐thermal efficiencies of winding linear Au nanostructures could be lower than those of straight linear Au nanostructures. Because the present black Au aqueous suspension contains variously deformed (winding and bent) linear Au nanostructures, theoretical studies suggest that the photo‐thermal efficiencies are slightly lower than those of the straight linear Au nanostructures. Nevertheless, their photo‐thermal efficiencies are sufficiently high compared with those of spherical nanoparticles and conventional nanorods with equivalent volumes.

### 2.3 Fabrication of Light Collecting Films with Black Au Nanostructures and Their Photo‐Thermal and Photo‐Thermoelectricity Performances

The aqueous suspensions containing the black Au nanostructures can be processed into a light‐collecting film. It is known that a metal‐dielectric structure is an efficient form for a solar collector.^[^ ^57^, ^58^, ^59^ ^]^ Most metal‐dielectric structures have been fabricated using inorganic materials. On the other hand, we attempted to prepare light‐collecting films by coating aqueous mixtures containing the black Au nanostructures and hydroxypropyl cellulose (HPC), a water‐soluble biopolymer, on various substrates (see the schematic in Figure 4D). HPC helped to keep the Au nanostructures exhibiting their own black characteristics even after drying. The concentration of the Au nanostructure was adjusted for the film to be suitable as a light collector for thermoelectricity (see Experimental Section for more details). We first prepared a film coated on a Cu plate. From Figure 4E, the black‐colored film (photograph) coated on the Cu surface showed a reflectance less than 5% at 400–2000 nm. The spectrum was comparable to that of the film prepared using HPC‐carbon black and sunselect, the commercially available sunlight‐collector film coated on Cu.

The HPC‐black Au nanostructure film can be prepared on transparent substrates, such as glass and flexible polymers. The sample showed nearly zero transmittance until a wavelength of 2000 nm (Figure 4F; see also photographs), and the reflectance was less than 7% (mostly 3–7%) at 400–2000 nm (Figure S13A, Supporting Information). There were no obvious differences in the transmittance and reflectance spectra of the films made of HPC‐carbon black and HPC‐black Au nanostructures. We also coated the HPC‐black Au nanostructures on a poly(ethylene terephthalate) (PET) film and laminated the HPC‐black Au nanostructures with a coating machine, as shown in Figure 4G (see Experimental Section for more details). Their transmittances were almost zero at 400–2000 nm (Figure 4H), and the reflectance of the HPC‐black Au nanostructure film on PET varied from 3–6%, and that of the HPC‐black Au nanostructure laminate film was less than 5% (Figure S13B, Supporting Information). The results demonstrate the processability of the black Au nanostructures for various applications.

Light‐collecting films for solar thermoelectric applications must have a high solar absorptance (*α*) (>0.9) and low emittance (*ε*) (<0.1). It is known that *α* can be estimated as follows:^[^ ^60^ ^]^

(1) $$\alpha \;=\int \emptyset_{\mathrm{sol}}\left(\lambda \right)A\left(\lambda \right)d\lambda /\int \emptyset_{\mathrm{sol}}\left(\lambda \right)d\lambda \;$$

where *ϕ*_sol_(*λ*) is the solar irradiance intensity at AM 1.5 G and *A*(*λ*) is the absorbance at the wavelength *λ*. For the film coated on the Cu substrate, *A* (*λ*) can be expressed as 1−reflectance (*λ*). We used 280–2000 nm for the calculations. *ε* can be estimated as follows:^[^ ^59^, ^60^ ^]^

(2) $$\varepsilon \;=\int P_{B}\left(\lambda \right)\varepsilon \left(\lambda \right)d\lambda /\int P_{B}\left(\lambda \right)d\lambda$$

where *P*_B_(*λ*) is the spectral radiance of a black body at a certain temperature *T* and *ε*(*λ*) is the emissivity of the material at the wavelength *λ*. According to Kirchhoff's law of thermal radiation,^[^ ^60^ ^]^ *ε*(*λ*) is equal to *A*(*λ*) in thermodynamic equilibrium. Again, *A*(*λ*) = 1−reflectance(*λ*) for the films coated on the Cu substrates. We measured the reflectance(*λ*) of the sample by using infrared spectroscopy from 2.5 to 25 µm (see Experimental Section for more details). It was estimated that the film contained HPC‐black Au nanostructures had *α*  of 0.97 and *ε*  of 0.08, and sunselect had *α* of 0.96 and *ε*  of 0.05.

**Figure 5:**
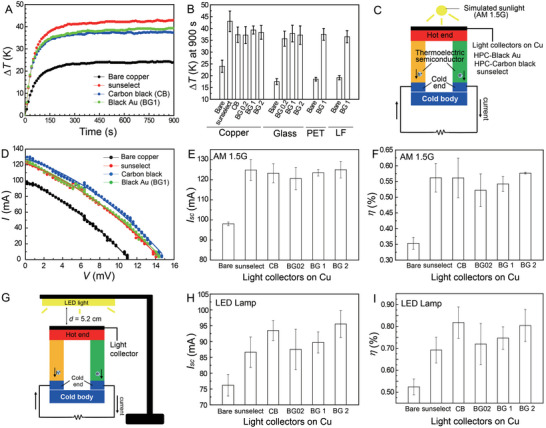
A) Temporal changes in the temperature (*∆T*) of the Cu plate, sunselect, and the HPC‐carbon black (CB) and HPC‐black Au nanostructure films on the Cu upon the illumination of the simulated sunlight (AM 1.5 G, 100 mW cm^−2^). B) The *∆T* (at 900 s) of the various light‐collecting films coated on various substrates and the HPC‐black Au nanostructure LF. The error bars indicate the standard deviation obtained from six independent measurements (*n* = 6). C) A schematic depicting the setup for the solar thermoelectricity experiments where the light collecting films coated on Cu were placed on the thermoelectricity device. D) *I*–*V* diagrams for the Cu plate, sunselect, and the Cu coated with HPC‐CB and HPC‐black Au nanostructure films. E) *I*_sc_ and F) *η* obtained from the thermoelectricity device (*I*–*V* diagrams) when using various light collecting films under the illumination of simulated sunlight (*n* = 3). G) A schematic depicting the setup in which the light‐collecting films coated on Cu were placed on the thermoelectricity device while illuminated by an LED light (*P*_light_ = 40 mW cm^−2^). H,I) *I*_sc_ and *η* obtained from the thermoelectricity device when using various light‐collecting films under the illumination of LED light (*n* = 3).

**Figure** **5**A shows the temporal change (*∆T*) in the temperatures of the Cu plate, sunselect, the HPC‐carbon black, and HPC‐black Au nanostructures under simulated sunlight illumination (AM 1.5 G, 100 mW cm^−2^). We also investigated the temperature changes of the other films, and Figure 5B summarizes *∆T* of all the samples tested after the illumination of simulated sunlight for 900 s. The Cu plate showed a *∆T* of 24 K, whereas the films prepared with HPC‐black Au nanostructures and HPC‐carbon black had*∆T* of  37–39 and 37 K, respectively. The sunselect had a *∆T* of 43 K. For the glass substrate, the glass slide had a *∆T* of 18 K, while the film prepared with HPC‐black Au nanostructures reached 36–38 K. The *∆T* of HPC‐Au nanostructures on PET and the HPC‐Au nanostructure laminate film were similar to those of films on glass.

We devised a thermoelectric experimental setup, as shown in Figure 5C. The film coated on the Cu substrate (1 × 1 cm^2^) was placed on the top surface of a thermoelectric generator (Bi_2_Te_3_), and its bottom surface was placed in contact with a cold body (an ice block) to keep the temperature difference between the hot and cold sides to generate electricity. After illumination with simulated sunlight for ≈100 s, current (*I*)–voltage (*V*) diagrams were recorded (Figure 5D), and the results are summarized in Figure 5E,F. The short‐circuit current *I*_sc_ (Figure 5E) was 98 ± 0.9 mA for the Cu plate, whereas those of the sunselect and the films prepared with HPC‐carbon black were 125 ± 5.3 and 123 ± 4.8 mA, respectively. The HPC‐black Au nanostructure film had an *I*_sc_ of 121 ± 5.5 to 125 ± 4.1 mA, respectively. The maximum power conversion efficiencies (*η*, %) (Figure 5F) were calculated as 100 × (*P*_max_/*P*_light_) where *P*_light_ is the incident light power (for simulated sunlight, *P*_light_ = 100 mW cm^−2^) and *P*_max_ is the maximum power produced by the thermoelectric device (from *I*–*V* profiles) during light illumination. *η* was 0.35 ± 0.02 for the Cu plate while the sunselect, the film coated with HPC‐carbon black, and HPC‐Au nanostructures had *η* of 0.56 ± 0.05, 0.56 ± 0.06, and 0.52 ± 0.05–0.58 ± 0.002%, respectively. It was reported that *η* reached 0.63% when using flat plate collectors and thermoelectric devices with ZnSb‐based alloys under the illumination of light (*P*_light_ of ≈82 mW cm^−2^).^[^ ^61^ ^]^ Therefore, the results under the present setup appeared close to the maximum efficiency for ordinary and everyday use. More importantly, the results demonstrate that the black Au nanostructures can exhibit thermoelectric performances comparable to those of the top‐class solar‐collecting commercial film.

We further investigated whether the present films are useful for harvesting low‐energy light from household appliances. A desk LED lamp was used, as shown in Figure 5G. The LED lamp produced a *P*_light_ of 40 mW cm^−2^ when the distance (*d*) between the lamp and the film was 5.2 cm. Although *I*_sc_ were lower than those obtained from the illumination of sunlight (Figure 5H), *η*s were enhanced (Figure 5I): the sunselect, the film coated with the HPC‐carbon black, and the HPC‐Au nanostructures had *η* of 0.69 ± 0.06 (maximum *η*: 0.77), 0.82 ± 0.07% (maximum *η*: 0.89), and 0.72 ± 0.09–0.80 ± 0.07% (maximum *η*: 0.90), respectively.

**Figure 6:**
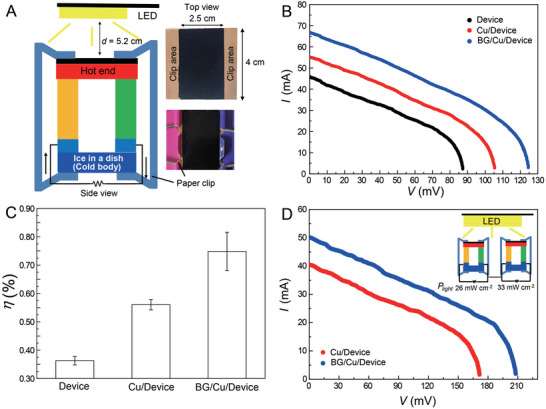
A) A schematic depicting the setup in which a 10 cm^2^ HPC‐black Au nanostructure film coated on Cu was placed on the thermoelectricity device while illuminated by LED light (*d* = 5.2 cm, *P*_light_ = 40 mW cm^−2^). B) *I–V* profiles for the device itself, the device joined to the Cu plate, and the device jointed to the Cu coated with black HPC‐Au nanostructure film. C) *η* obtained from the thermoelectricity device for the three cases shown in (B) with the setup in (A). The error bars indicate the standard deviation obtained from three independent measurements (*n* = 3). D) *I–V* profiles obtained from an experimental setup where the two thermoelectricity devices were connected in series (see inset cartoon): each device is placed on a 10 cm^2^ black HPC‐Au nanostructure film coated on Cu. *P*_light_ delivered to each device were 26 and 33 mW cm^−2^, respectively. For the preparation of black HPC‐Au nanostructure film, we used the black Au suspensions synthesized by using the tri‐sodium citrate and 1 wt% PVP.

We introduced a larger‐area thermoelectric device (4 × 4 cm^2^) to generate a larger amount of power, as schematically shown in **Figure** **6**A. In this case, it was necessary to use paper clips for close contact between the ice block (made from a refrigerator) and the device. The clips also help maintain intimate contact between the device and the light‐collecting film placed on the device. The LED light illuminated 10 cm^2^ of the Cu plate or the HPC‐black Au nanostructure film coated on the Cu after clipping. We compared the photo‐thermoelectricity performances among the clip‐attached device (without the Cu plate and film), the clip‐attached device with the Cu plate, and the clip‐attached device with the HPC‐black Au nanostructure film on the Cu. From Figure 6B,C, the device itself produced an *I*_sc_ of 45 ± 1.5 mA, a *V*_oc_ of 83 ± 1.0 mV, and a *η* of 0.36 ± 0.01%. The device with the Cu plate had an *I*_sc_ of 56 ± 0.7 mA, a *V*_oc_ of 104 ± 1.1 mV, and a *η* of 0.56 ± 0.02%. The device with the HPC‐black Au nanostructure film on Cu had *I*_sc_ of 64 ± 3.5 mA, *V*_oc_ of 123 ± 4.5 mV, and *η* of 0.75 ± 0.07%. These results demonstrate the possibility that the LED light used in the house can be effectively converted into electricity using a thermoelectric device when introducing the HPC‐black Au nanostructure film. Last, we connected the two thermoelectric devices in series to increase *V*_oc_ (Figure 6D). As shown in the inset, each device was illuminated by the light with a *P*_light_ of 26 and 33 mW cm^−2^. As a result, the serially connected device equipped with the black Au nanostructures had a lower *I*_sc_ of 50 ± 0.76 mA compared with the *I*_sc_ from the setup shown in Figure 6A. However, *V*_oc_ increased to 205 ± 3.3 mV. *η* reached 0.65 ± 0.03% (for the Cu plate *I*_sc_ = 41 ± 1.90 mA, *V*_oc_ = 167 ± 6.2 mV, *η* = 0.44 ± 0.03%).

## 3 Conclusion

We demonstrated that the present linear black Au nanostructures can absorb considerable amounts of sunlight and low‐energy indoor light to produce heat, thereby contributing to electric generation through a photo‐thermoelectricity system. The linear black Au nanostructures could be simply synthesized in aqueous solutions, and they can also be readily processed by combining them with natural polymers to form metal‐dielectric light‐collecting films. The films had high *α* (0.97) and low *ε* (0.08), which are the important parameters of the films for solar thermoelectricity. Therefore, the film exhibits comparable photo‐thermal and photo‐thermoelectric performances with a top‐class solar light collecting film under simulated sunlight and indoor LED light illumination. In addition, we demonstrate that black Au nanostructures can be fabricated in various dimensions for photo‐thermoelectric systems to produce more electricity. Therefore, more usable energy is expected to be generated if photo‐thermoelectric device systems are optimized to harvest low‐energy light and when the light‐collecting films are fabricated to meet the optimized systems. We also demonstrated that the film can be formed on a flexible film or laminated, making it potentially useful as a flexible photo‐thermoelectric system. Moreover, the black Au nanostructures could be processed into various forms for other photo‐thermal (such as steam generation for desalination/water purification) or photo‐electricity purposes. In conclusion, the current linear black Au nanostructures may have a significant impact not only on the usage of photo‐thermoelectric devices to utilize low‐energy indoor lighting, but also on other light‐associated applications.

## 4 Experimental Section

### Materials

HAuCl_4_∙3H_2_O (99.9%), PVP (MW ≈ 55 000), and HPC (MW ≈ 1 00 000) were purchased from Sigma Aldrich. Tri‐sodium citrate (99%) was purchased from Fisher Scientific. Glass slide and PET films (thickness: 100 µm) were obtained from Paul Marienfeld GmbH & Co. (Germany) and Lamiace (Republic of Korea), respectively. A laminating film made from PET, polyethylene, and ethylene‐vinyl acetate copolymer was obtained from Hyundai office (Republic of Korea). The sunselect was obtained from Alanod‐Solar GmbH & Co. KG (Germany).

### Synthesis of Black Au Nanostructures

Typically, 0.3 mL of 25 mm HAuCl_4_ aqueous solution was added to 5 mL aqueous solution containing 0.3 m tri‐sodium citrate and 0.2–2 wt% PVP. After the reaction was conducted for 5 min at room temperature (20–25 °C), the aqueous suspension was centrifuged at 11 000 rpm for 30 min. After removing the supernatant, the cake was re‐dispersed in deionized water.

### Black Au Nanostructure Film Preparations

The black Au nanostructure aqueous suspension was centrifuged again, and a 2 wt% HPC aqueous solution was added to the Au nanostructure cake. The amount of HPC aqueous solution was adjusted to increase the concentration of the Au nanostructures by 150‐fold from the black Au aqueous suspension with an approximate extinction of 0.5 at 520 nm. The volume of the HPC‐black Au nanostructure aqueous suspension added to the substrate depended on the area of the substrate. For example, 0.4 mL per 1.5 × 1.5 cm^2^ of HPC‐black Au nanostructure aqueous suspension was added to Cu, glass, and PET, and the coating films were dried at 50 °C for 45 min in a convection oven. For the preparation of the HPC‐black Au nanostructure laminate film (see Figure 4G), 0.4 mL per 1.5 × 1.5 cm^2^ of HPC‐black Au nanostructure aqueous suspension was added to one side of a commercial flexible laminating film. After drying, the other film covered the HPC‐black Au nanostructure film, and the overlapped films were transferred to a laminating machine (Hyundai office, Republic of Korea). After cutting the sides, an HPC‐black Au nanostructured laminate film was obtained.

### Characterizations

The morphology of the Au nanostructures was observed using a TEM (JEM2100F, JEOL, Japan). The extinction spectra of the aqueous suspension containing black Au nanostructures were measured using a UV–vis‐NIR spectrophotometer (V‐670, JASCO, Japan). The transmittance and reflectance of the films coated on various substrates (including the laminated films) were measured using the same instrument equipped with a 60‐mm integrating sphere (ISN‐723) and a Spectralon reflectance standard (Labsphere). For the calculation of the emittance (*ε*) of the films on Cu, we used an infrared spectroscopy (NICOLET IS50, Thermo Fischer Scientific, USA) under the attenuated total reflection mode. The background signals were calibrated from 2.5 to 25 µm using an Au (100 nm)‐coated silicon wafer and a Cu plate, and the reflectance of the sunselect and the HPC‐black Au nanostructure film on the Cu were recorded. The absorbance *A*(*λ*) of each film was obtained using the relationship 1−reflectance(*λ*) for the films coated on the Cu substrates, and *A*(*λ*) was used to calculate *ε* using Equation (2). The temperature increment was measured by attaching a thermocouple to the back side of the light‐collecting films during the incidence of simulated sunlight from a solar simulator (Model 10 500, Abet Technologies, USA) under AM 1.5 G conditions. The thermoelectric current (*I*) and voltage (*V*) profiles generated from a thermoelectric device (Bi_2_Te_3_ with a figure of merit *ZT* of 1.1 for *p*‐type and 1.0 for *n*‐type at 300 K, EVERREDtronics, China) were recorded by connecting the thermoelectric device to a potentiostat (WMPG1000, WonATech, Republic of Korea). The experimental setup is shown in Figures 5C,G and 6A,D. Depending on the sample area (1 or 10 cm^2^), we used thermoelectric devices with different areas (1 and 16 cm^2^, respectively). For the experiments where the LED light illuminated the light‐collecting films, we used a desk LED lamp (Solarzen, wavelength: 400–750 nm, Republic of Korea).

### Calculations

COMSOL Multiphysics (Version 5.4, Waveoptics module, Sweden) software was used to investigate the theoretical extinction and absorption cross sections of the black Au nanostructures. For the refractive index of water, we used 1.33. The complex refractive indices of Au were adapted from the experimental values provided by a literature.^[^ ^62^ ^]^ The incident electric‐field intensity was 1 V m^−1^. Linearly polarized electromagnetic waves were incident from the three different directions to the straight linear and bent Au nanostructures (see Figure 3D). For each direction, electric (*E*) and magnetic (*H*) fields were polarized along the two directions (e.g., *E*_z_, *E*_y_ and *H*_y_, *H*_z_ for *k_x_* where *k_x_* is the propagation vector heading *x*‐direction). Therefore, it was possible to obtain the optical spectra of the Au nanostructures without the effect of polarization directions. It was assumed that a straight linear Au nanostructure was formed by the two hemispheres attached to both ends of a cylinder. It was also assumed that the bent Au nanostructures were made by connecting two cylinders to both ends of a torus segment and sequentially attaching the hemispheres to the ends of the cylinders. We assumed that the winding structure was constructed by combining the torus segments (bent segments with bent angles of 13, 17, 27, and 40°), cylinders (straight segments), and hemispheres.

The heat transfer module of COMSOL Multiphysic*s* was used for the photo‐thermal study. Incident power of 1 mW cm^−2^ was applied to the Au nanostructures. The volume fraction of Au nanostructures in the surrounding medium (water) was 1.3 × 10^−4^. The temperature change was obtained at the center (coordinate: (0,0,0)) of the simulation geometry.

### Statistical Analysis

This work presented some data as the average ± standard deviation from a number of independent instrumental measurements for each sample. The number of measurements (*n*) was six for the studies on the temperature changes while illuminating the simulated sunlight on the light‐collecting films having the areas of either 1 cm^2^, and *n* = 3 for the photo‐thermoelectricity performance while illuminating the simulated sunlight and the LED light on the light‐collecting films having the areas of either 1 or 10 cm^2^ (including the two samples connected in series).

## Conflict of Interest

The authors declare no conflict of interest.

## Supporting information

Supporting Information

## Data Availability

Research data are not shared.

## References

[1] X. Cao , X. Dai , J. Liu , Energy Build. 2016, 128, 198.

[2] P. M. Ushasree , B. Bora , Solar Energy Capture Materials, Royal Society of Chemistry, London, 2019.

[3] S. Günes , H. Neugebauer , N. S. Sariciftci , Chem. Rev. 2007, 107, 1324. 17428026 10.1021/cr050149z

[4] M. A. Green , S. Pillai , Nat. Photonics 2012, 6, 130.

[5] C. Clavero , Nat. Photonics 2014, 8, 95.

[6] M. Gratzel , J. Photochem. Photobiol. 2003, 4, 145.

[7] M. T. Sheldon , J. van de Groep , A. M. Brown , A. Polman , H. A. Atwater , Science 2014, 346, 828. 25395532 10.1126/science.1258405

[8] J. Chen , Z. Ye , F. Yang , Y. Yin , Small Sci. 2021, 1, 2000055. 10.1002/smsc.202000055 PMC11935886 40212468

[9] D. G. Baranov , Y. Xiao , I. A. Nechepurenko , A. Krasnok , A. Alù , M. A. Kats , Nat. Mater. 2019, 18, 920. 31133732 10.1038/s41563-019-0363-y

[10] Z. Wang , W. Yang , F. Qiu , X. Zhang , X. Zhao , Renewable Sustainable Energy Rev. 2015, 41, 68.

[11] K. Yaman , G. Arslan , J. Renewable Sustainable Energy 2018, 10, 023703.

[12] H. El Ferouali , A. Zoukit , I. Salhi , T. El Kilali , S. Doubabi , N. Abdenouri , J. Renewable Sustainable Energy 2018, 10, 043709.

[13] L. Zhou , Y. Tan , D. Ji , B. Zhu , P. Zhang , J. Xu , Q. Gan , Z. Yu , J. Zhu , Sci. Adv. 2016, 2, e1501227. 27152335 10.1126/sciadv.1501227 PMC4846456

[14] M. Gao , C. K. Peh , H. T. Phan , L. Zhu , G. W. Ho , Adv. Energy Mater. 2018, 8, 1800711.

[15] F. Zhao , X. Zhou , Y. Shi , X. Qian , M. Alexander , X. Zhao , S. Mendez , R. Yang , L. Qu , G. Yu , Nat. Nanotechnol. 2018, 13, 489. 29610528 10.1038/s41565-018-0097-z

[16] Q. F. Guan , Z. M. Han , Z. C. Ling , H. B. Yang , S. H. Yu , Nano Lett. 2020, 20, 5699. 32638594 10.1021/acs.nanolett.0c01088

[17] L. L. Baranowski , G. J. Snyder , E. S. Toberer , Energy Environ. Sci. 2012, 5, 9055.

[18] G. Chen , J. Appl. Phys. 2011, 109, 104908.

[19] D. Kraemer , B. Poudel , H. P. Feng , J. C. Caylor , B. Yu , X. Yan , Y. Ma , X. W. Wang , D. Z. Wang , A. Muto , Nat. Mater. 2011, 10, 532. 21532584 10.1038/nmat3013

[20] M. Zhang , L. Miao , Y. P. Kang , S. Tanemura , C. A. Fisher , G. Xu , X. Chen , Z. Guang , Appl. Energy 2013, 109, 51.

[21] V. Rinnerbauer , A. Lenert , D. M. Bierman , Y. X. Yeng , W. R. Chan , R. D. Geil , J. J. Senkevich , J. D. Joannopoulos , E. N. Wang , M. Soljačić , I. Celanovic , Adv. Energy Mater. 2014, 4, 1400334.

[22] D. Kraemer , Q. Jie , K. McEnaney , F. Cao , W. Liu , L. A. Weinstein , J. Loomis , J. Ren , Z. Chen , G. Chen , Nat. Energy 2016, 1, 16153.

[23] J. P. Jurado , B. Dorling , O. Zapata‐Arteaga , A. Roig , A. Mihi , M. Campoy‐Quiles , Adv. Energy Mater. 2019, 9, 1902385.

[24] C. Chang , Z. Wang , B. Fu , Y. Ji , Sci. Rep. 2020, 10, 20500. 33235267 10.1038/s41598-020-77442-y PMC7687880

[25] C. M. Cobley , J. Chen , E. C. Cho , L. V. Wang , Y. Xia , Chem. Soc. Rev. 2011, 40, 44. 20818451 10.1039/b821763g

[26] X. Liu , L. Li , Y. Yang , Y. Yin , C. Gao , Nanoscale 2014, 6, 4513. 24658147 10.1039/c4nr00254g

[27] E. C. Cho , C. Kim , F. Zhou , C. M. Cobley , K. H. Song , J. Chen , Z.‐Y. Li , L. V. Wang , Y. Xia , J. Phys. Chem. C 2009, 113, 9023. 10.1021/jp903343p PMC2695596 19680423

[28] D. Liu , F. Zhou , C. Li , T. Zhang , H. Zhang , W. Cai , Y. Li , Angew. Chem., Int. Ed. 2015, 54, 9596. 10.1002/anie.201503384 26111204

[29] M. ElKabbash , A. Sousa‐Castillo , Q. Nguyen , R. Mariño‐Fernández , N. Hoffman , M. A. Correa‐Duarte , G. Strangi , Adv. Opt. Mater. 2017, 5, 1700617.

[30] N. Kwon , H. Oh , R. Kim , A. Sinha , J. Kim , J. Shin , J. W. M. Chon , B. Lim , Nano Lett. 2018, 18, 5927. 30075632 10.1021/acs.nanolett.8b02629

[31] R. Wadhwa , K. K. Yadav , T. Goswami , Ankush , S. K. Guchhat , Sunaina , S. T. Nishanthi , H. N. Ghosh , M. Jha , ACS Appl. Mater. Interfaces 2021, 13, 9942. 33606504 10.1021/acsami.0c21010

[32] Q. Zhong , J. Feng , B. Jiang , Y. Fan , Q. Zhang , J. Chen , Y. Yin , J. Am. Chem. Soc. 2021, 143, 20513. 34812625 10.1021/jacs.1c11242

[33] M. Dhiman , M. Ayan , A. Das , R. Belgamwar , B. Chalke , Y. Lee , K. Sim , J.‐M. Nam , V. Polshettiwar , Chem. Sci. 2019, 10, 6594. 31367310 10.1039/c9sc02369k PMC6625417

[34] Y. Liu , Z. Liu , Q. Huang , X. Liang , X. Zhou , H. Fu , Q. Wu , J. Zhang , W. Xie , J. Mater. Chem. A 2019, 7, 2581.

[35] K. Bae , G. Kang , S. K. Cho , W. Park , K. Kim , W. J. Padilla , Nat. Commun. 2015, 6, 10103. 26657535 10.1038/ncomms10103 PMC4682046

[36] C. Ng , L. W. Yap , A. Roberts , W. Cheng , D. E. Gomez , Adv. Funct. Mater. 2017, 27, 1604080.

[37] R. Yu , J. Wang , M. Han , M. Zhang , P. Zeng , W. Dang , Z. Tian , ACS Omega 2020, 5, 8293. 32309740 10.1021/acsomega.0c00698 PMC7161050

[38] T. Søndergaard , S. M. Novikov , T. Holmgaard , R. L. Eriksen , J. Beermann , Z. Han , K. Pedersen , S. I. Bozhevolnyi , Nat. Commun. 2012, 3, 969. 22828629 10.1038/ncomms1976

[39] J. Zheng , X. Cheng , H. Zhang , X. Bai , R. Ai , L. Shao , J. Wang , Chem. Rev. 2021, 121, 13342. 34569789 10.1021/acs.chemrev.1c00422

[40] H. ‐H. Chang , C. J. Murphy , Chem. Mater. 2018, 30, 1427. 31404258 10.1021/acs.chemmater.7b05310 PMC6688645

[41] Z. Li , J. Tao , X. Lu , Y. Zhu , Y. Xia , Nano Lett. 2008, 8, 3052. 18681484 10.1021/nl8017127

[42] R. Takahata , S. Yamazoe , K. Koyasu , K. Imura , T. J. Tsukuda , J. Am. Chem. Soc. 2018, 140, 6640. 29694041 10.1021/jacs.8b02884

[43] Y. N. Xia , Y. J. Xiong , B. Lim , S. E. Skrabalak , Angew. Chem., Int. Ed. 2009, 48, 60. 10.1002/anie.200802248 PMC2791829 19053095

[44] K. I. Requejo , A. V. Liopo , P. J. Derry , E. R. Zubarev , Langmuir 2017, 33, 12681. 29032680 10.1021/acs.langmuir.7b02942

[45] C. C. Li , L. B. Chen , Q. H. Li , T. H. Wang , CrystEngComm 2012, 14, 7549.

[46] J. Zhang , H. Liu , Z. Wang , N. Ming , Adv. Funct. Mater. 2007, 17, 3295.

[47] Y. Zhai , J. S. DuChene , Y. C. Wang , J. Qiu , A. C. Johnston‐Peck , B. You , W. Guo , B. DiCiaccio , K. Qian , E. W. Zhao , F. Ooi , D. Hu , D. Su , E. A. Stach , Z. Zhu , W. D. Wei , Nat. Mater. 2016, 15, 889. 27376686 10.1038/nmat4683

[48] B.‐K. Pong , H. I. Elim , J.‐X. Chong , W. Ji , B. L. Trout , J.‐Y. Lee , J. Phys. Chem. C 2007, 111, 6281.

[49] L. Pei , K. Mori , M. Adachi , Langmuir 2004, 20, 7837. 15323538 10.1021/la049262v

[50] R. M. Sarhan , S. Kogikoski Jr , R. M. Schürmann , Y. Zhao , A. Krause , B. Schmidt , I. Bald , Y. Lu , *ChemRxiv*, 2022. This content is a preprint and has not been peer‐reviewed; 10.26434/chemrxiv-2022-mscd9-v2.

[51] S. Navaladian , C. M. Janet , B. Viswanathan , T. K. Varadarajan , R. P. Viswanath , J. Phys. Chem. C 2007, 111, 14150.

[52] C. D. Chen , Y. T. Yeh , C. R. C. Wang , J. Phys. Chem. Solids 2001, 62, 1587.

[53] S. Link , M. B. Mohamed , M. A. El‐Sayed , J. Phys. Chem. B 1999, 103, 3073.

[54] C. Enkrich , M. Wegener , S. Linden , S. Burger , L. Zschiedrich , F. Schmidt , J. F. Zhou , T. Koschny , C. M. Soukoulis , Phys. Rev. Lett. 2005, 95, 5. 10.1103/PhysRevLett.95.203901 16384056

[55] C. Cao , J. Zhang , X. Wen , S. L. Dodson , N. T. Dao , L. M. Wong , S. Wang , S. Li , A. T. Phan , Q. Xiong , ACS Nano 2013, 7, 583. 10.1021/nn401645t 23952283

[56] A. Babynina , M. Fedoruk , P. Kühler , A. Meledin , M. Döblinger , T. Lohmüller , Nano Lett. 2016, 16, 6485. 27598653 10.1021/acs.nanolett.6b03029

[57] M. Farooq , A. A. Green , M. G. Hutchins , Sol. Energy Mater. Sol. Cells 1998, 54, 67.

[58] G. E. McDonald , Sol. Energy 1975, 17, 119.

[59] D. H. Lee , S. B. Pyun , Y. Bae , D. P. Kang , J.‐W. Park , E. C. Cho , ACS Appl. Mater. Interfaces 2017, 9, 43583. 29172424 10.1021/acsami.7b11446

[60] J. A. Duffie , W. A. Beckman , Solar Engineering of Thermal Processes, John Wiley & Sons, Hoboken, NJ, 2013.

[61] M. Telkes , J. Appl. Phys. 1954, 25, 765.

[62] Johnson, P. B. , R. W. Christy , Phys. Rev. B 1972, 6, 4370.

