# Supplementary material for: Synthesis of Linear Black Gold Nanostructures Processable as Sunlight and Low‐Energy Light Collecting Films for Photo‐Thermoelectricity
Source: Adv Sci (Weinh). 2023 Feb 24;10(13):2207415. doi: 10.1002/advs.202207415 (PMC10161013; doi:10.1002/advs.202207415)
Supplement: Supplementary file 1 — Supporting Information [file ADVS-10-2207415-s001.pdf]

ADVANCED SCIENCE
Open Access

Supporting Information

for *Adv. Sci.*, DOI 10.1002/advs.202207415

Synthesis of Linear Black Gold Nanostructures Processable as Sunlight and Low-Energy Light Collecting Films for Photo-Thermoelectricity

*Jeong Han Kim, Seung Beom Pyun, Min Ju Choi, Ji Won Yeon, Young Ji Hwang and Eun Chul Cho**

Supporting Information

Synthesis of Linear Black Gold Nanostructures Processible as Sunlight and Low-Energy Light Collecting Films for Photo-Thermoelectricity

Jeong Han Kim[†], *Seung Beom Pyun*[†], *Min Ju Choi, Ji Won Yeon, Young Ji Hwang, and Eun Chul Cho*[*]

Department of Chemical Engineering, Hanyang University, Seoul, 04763, Republic of Korea.

Corresponding author: [*]E-mail: enjoe@hanyang.ac.kr

[†]: J.H.K. and S.B.P. contributed equally to this work.

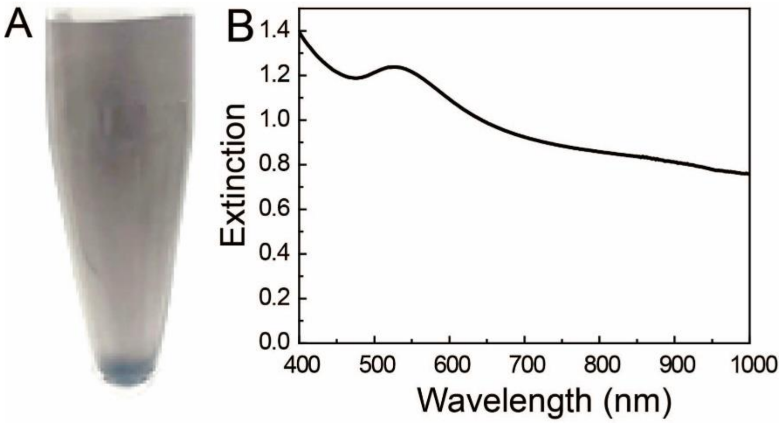

Figure S1. (A) A photograph of an aqueous suspension obtained after the centrifugation of a reaction mass which was formed after addition of 0.3 mL of 25 mM $HAuCl_4$ aqueous solution to 5 mL aqueous solution containing 0.3 M tri-sodium citrate and 2 wt% of PVP. The reaction was stopped at t = 5 min, and centrifugation was conducted for 30 min at 11000 rpm. (B) The corresponding UV-VIS-NIR extinction spectra of the supernatant shown in (A).

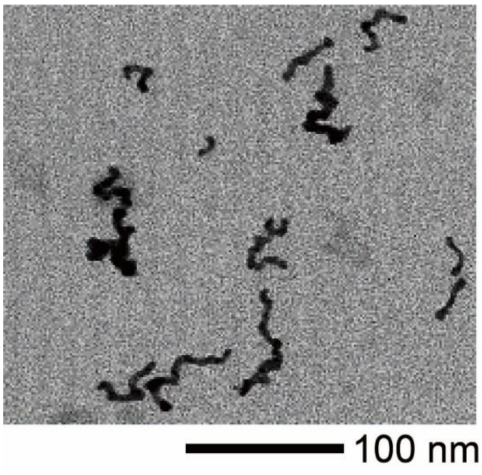

Figure S2. A TEM image of the Au nanostructures observed from the black Au aqueous suspension synthesized by using the aqueous solution containing tri-sodium citrate and 2 wt% of PVP.

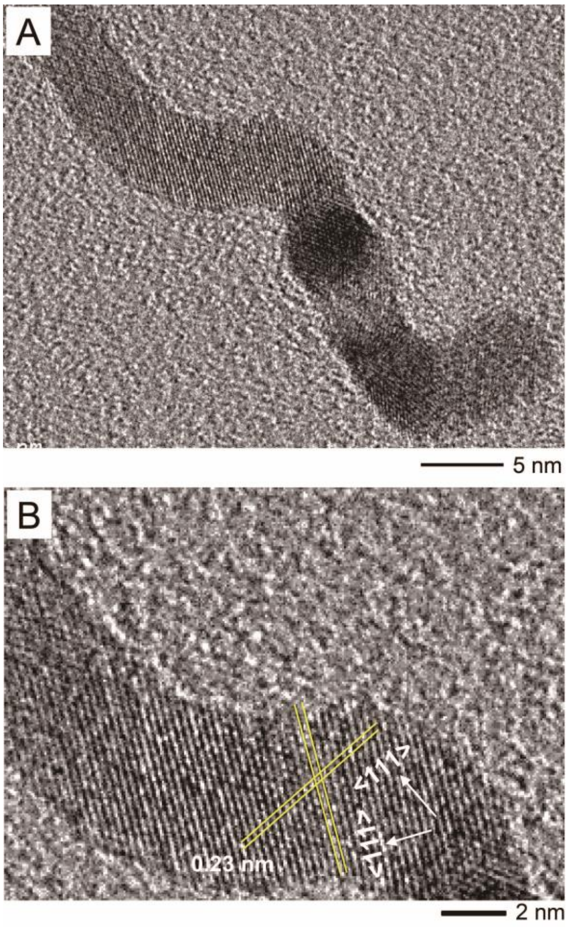

Figure S3. (A) A TEM image of the Au nanostructure observed from the black Au aqueous suspension synthesized by using the aqueous solution containing tri-sodium citrate and 0.2 wt% of PVP. (B) A magnified TEM image of (A), along with the analysis of lattice structure.

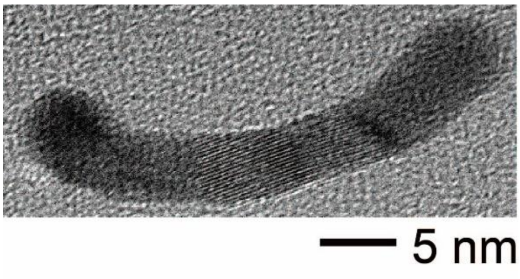

Figure S4. A TEM image of the Au nanostructure observed from the black Au aqueous suspension synthesized by using the aqueous solution containing tri-sodium citrate and 1 wt% of PVP.

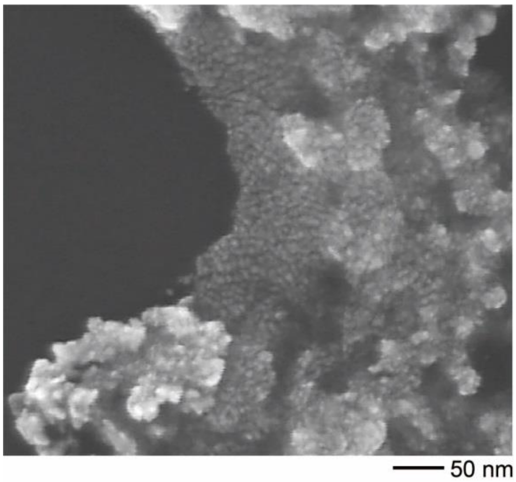

Figure S5. A scanning electron microscopy (S-4800, HITACHI, Japan) image of the Au nanostructure synthesized after addition of 0.3 mL of 25 mM $HAuCl_4$ aqueous solution to 5 mL aqueous solution containing 0.3 M tri-sodium citrate (without using PVP). Tiny Au nanoparticles were aggregated.

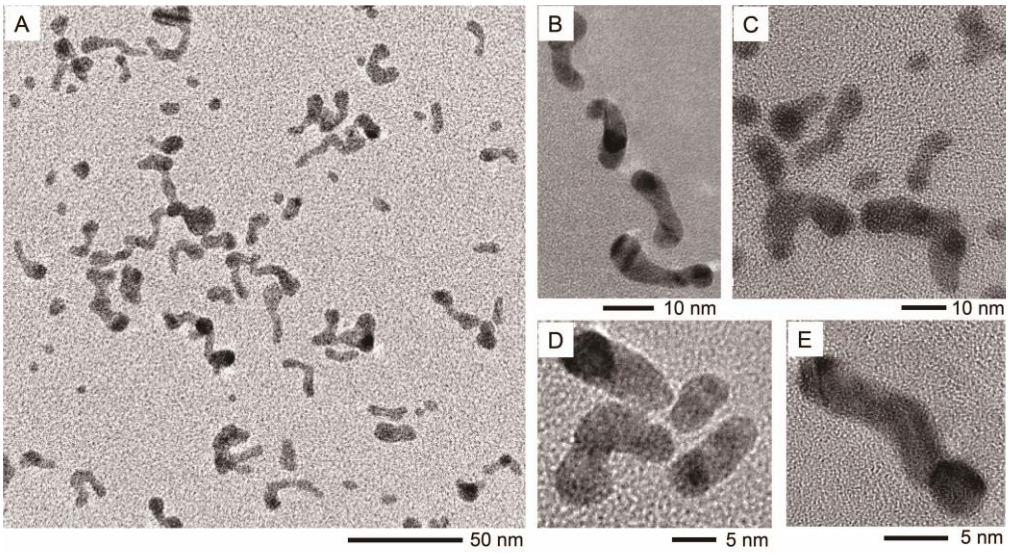

Figure S6. TEM images of the Au nanostructures synthesized by using the aqueous solution containing tri-sodium citrate and 0.1 wt% of PVP.

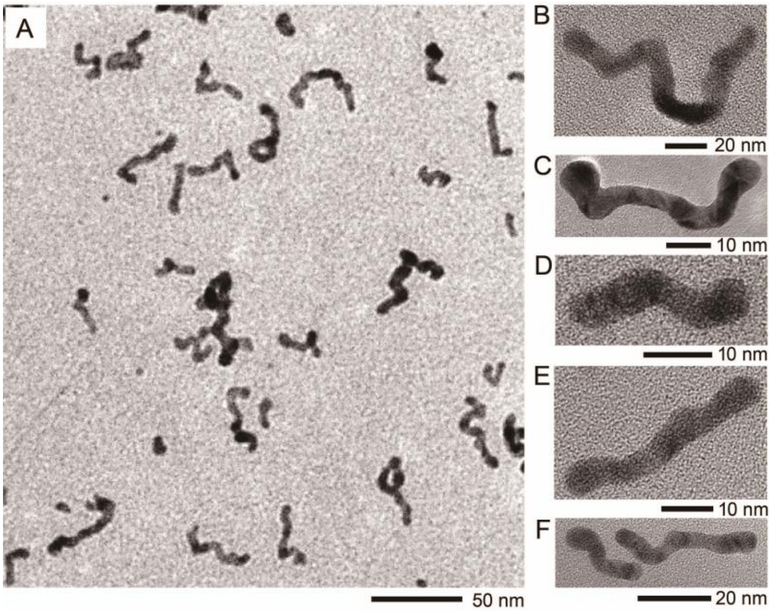

Figure S7. TEM images of the Au nanostructures synthesized by using the aqueous solution containing tri-sodium citrate and 5 wt% of PVP.

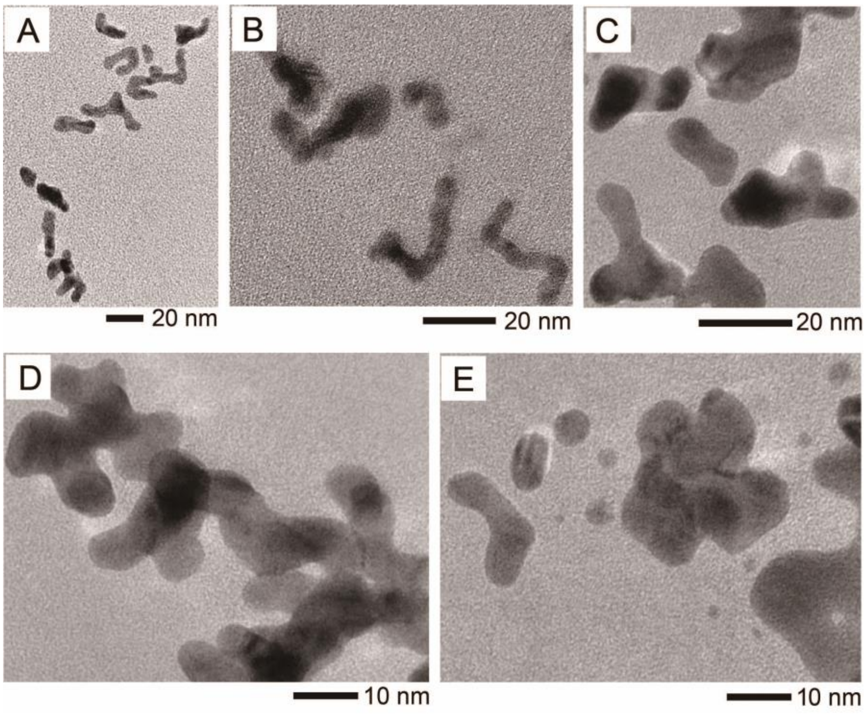

Figure S8. TEM images of the Au nanostructures synthesized by using the aqueous solution containing tri-sodium citrate and 10 wt% of PVP.

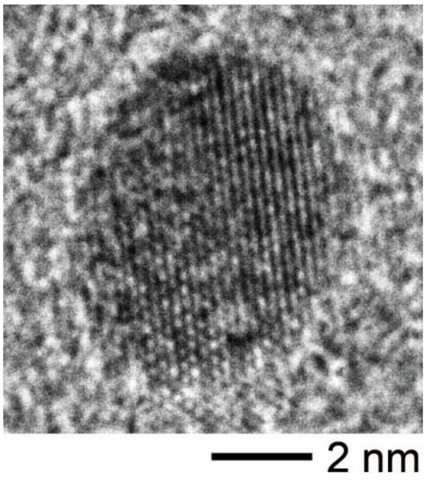

Figure S9. A TEM image of an Au nanoparticle observed from the black Au aqueous suspension synthesized by using the aqueous solution containing tri-sodium citrate and 1 wt% of PVP.

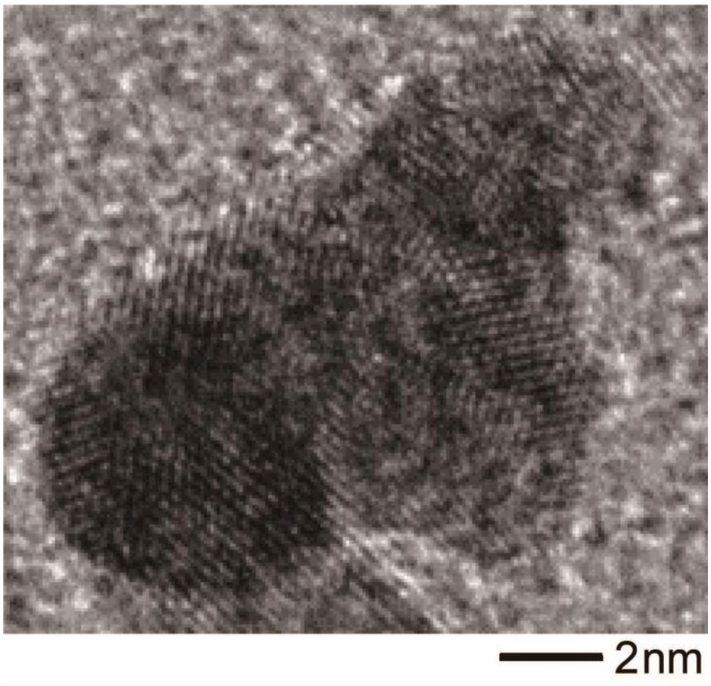

Figure S10. A magnified TEM image of the Au nanostructure shown in Figure 2J which was observed from the black Au aqueous suspension by using the aqueous solution containing tri-sodium citrate and 0.2 wt% of PVP.

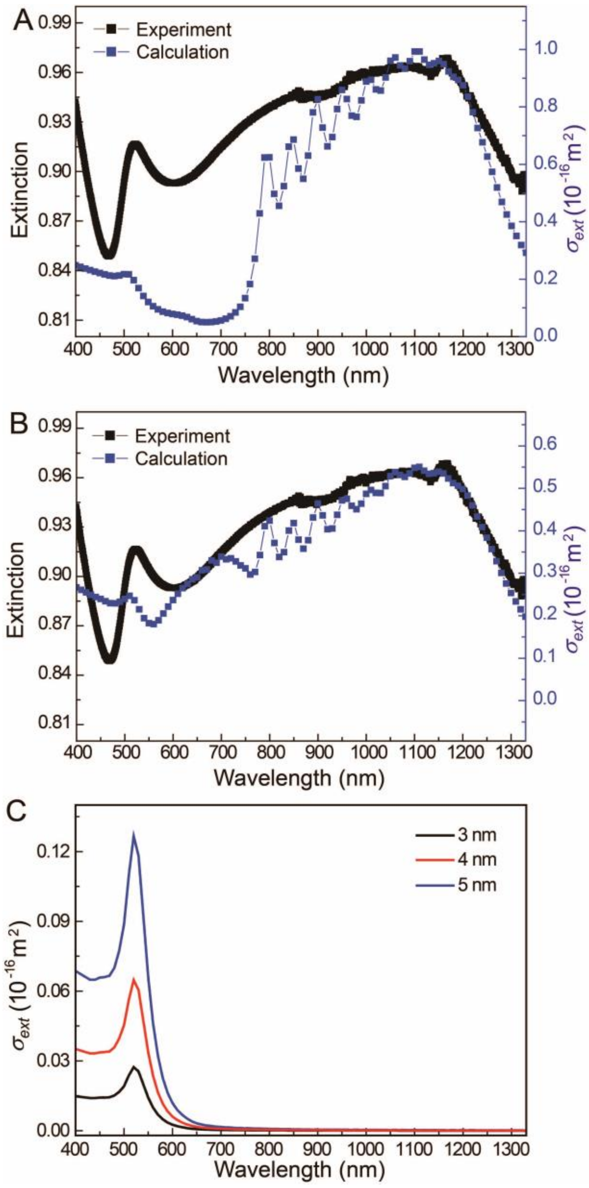

Figure S11. (A) A fitting result (blue squares) to the experimental data (black spheres) of the black Au aqueous suspension synthesized by using the aqueous solution containing tri-sodium citrate and 2 wt% of PVP. For the calculation, we only consider the straight linear Au nanostructures (bent angle: 0°) with the AR of 4–10. (B) A fitting result (blue squares) to the experimental data (black spheres) of the black Au aqueous suspension by considering the bent Au nanostructures (bent angles 0–180°) with the AR of 4–10. For the calculated data in (B), we assumed that the equal number of the Au nanostructures with bent angles from 0 to 180° existed for each AR. (C) Calculated extinction cross-sections (σ_{ext}) spectra for the spherical Au nanoparticles having the diameters of 3, 4, and 5 nm.

Table S1. Calculated volume fraction (%) of the linear Au nanostructures and spherical nanoparticles after the fitting the calculated spectra to the experimental spectra of the black Au aqueous suspensions.

PVP concentration		1 wt%	2 wt%
Au nanostructure Aspect Ratio	4	4	10.4
	4.5	4.5	8.7
	5	5.1	9.4
	5.5	6.1	8.8
	6	7.3	8.3
	6.5	8.6	8.6
	7	5.9	8.2
	7.5	6.4	7.2
	8	3.8	7.1
	8.5	4.1	3.8
	9	0	1.3
	9.5	0	0.7
	10	0	0.7
	Total	55.8	83.2
Au Nanospheres (3–5 nm)		44.2	16.8

Note: For the best fitting to the black Au aqueous suspension synthesized by using the aqueous solution containing tri-sodium citrate and 2 wt% of PVP, we assumed that the equal number of the Au nanostructure with bent angles from 0 to 180° existed for each AR. For the best fitting to the black Au aqueous suspension synthesized by using the aqueous solution containing tri-sodium citrate and 1 wt% PVP, we considered the Au nanostructure with bent angles from 0 to 90°. We assumed that the equal number of the Au nanostructure with bent angles from 0 to 90° existed for each AR.

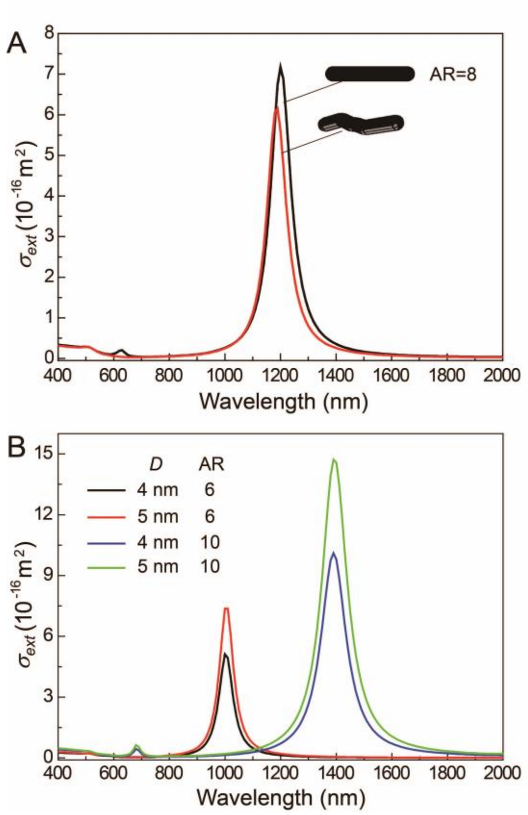

Figure S12. (A) σ_{ext} spectra of the straight and winding linear Au nanostructures with AR=8. (B) σ_{ext} spectra of the straight linear Au nanostructures having the diameter of 4 and 5 nm for the AR=6 and 10.

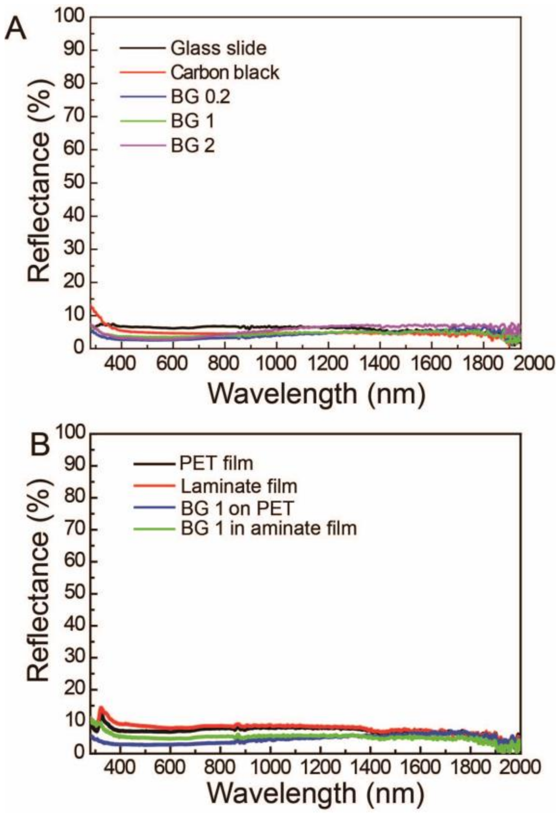

Figure S13. (A) Reflectance spectra of the glass slide and various light collecting films coated on the glass substrate (B) Reflectance spectra of the PET and laminating films, HPC-black Au nanostructure film on PET, and HPC-black Au nanostructures in laminate film.
